# Supplementary material for: NMR Spectroscopy for the Validation of AlphaFold2 Structures
Source: bioRxiv. 2025 Feb 8:2025.02.04.636507. Preprint. [Version 1] doi: 10.1101/2025.02.04.636507 (PMC11838581; doi:10.1101/2025.02.04.636507)
Supplement: Supplement 1 [file media-1.pdf]

Supplemental Information for NMR Spectroscopy for the Validation of AlphaFold2 Structures

Jake Williams[1*], Isabelle A. Gagnon[3] and Joseph R. Sachleben[2*]

[1*]Department of Computer Science, University of Chicago, Chicago, IL.
[3]Department of Biochemistry and Molecular Biology, University of Chicago, Chicago, IL.
[2*]Biomolecular NMR Facility, University of Chicago, Chicago, IL.

*Corresponding author(s). E-mail(s): williamsjl@uchicago.edu; jsachleben@uchicago.edu;
Contributing authors: igagnon@uchicago.edu;

1 Additional Training Details

In this section, we provide further details on training our support vector machines. Calculating CS and DS require values for the parameters ϵ_{CS} and ϵ_D. ϵ_{CS} and ϵ_D are Cα distances that provide cutoffs in the calculation of CS and DS. They were determined by a small grid search over a realistic range of parameters. For the distance score cutoff value, ϵ_D, we calculated the correlation between DS and the TM-score over the training data for various values of ϵ_D, see Figure 1. We expect this correlation between DS and TM-score to be negative, so we selected the value of ϵ_D that gives the greatest negative correlation. As seen in Figure 1, the best performing value was $\epsilon_D = 10$ Å.

Similarly, for the contact score threshold ϵ_{CS}, we performed a grid search over the values 6, 9, 12, and 15 Å, and measured the correlation between the contact heuristic and the TM-score to find that 12 Å was optimal, Figure 2. These grid searches were performed before the full dataset had been collected and without the mismatched data. We chose to use these values for later calculations using both the original and augmented datasets to prevent over fitting of the parameters.

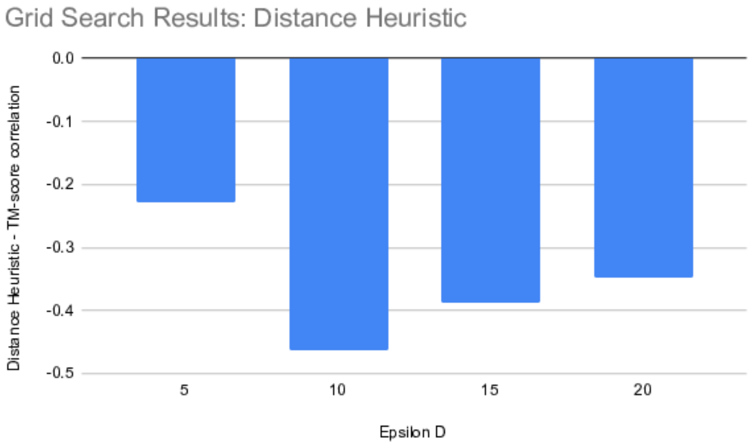

Fig. 1: Grid search over the distance score cutoff values shows that $\epsilon_D = 10$ Angstroms provides the greatest negative correlation between the distance heuristic and TM-score.

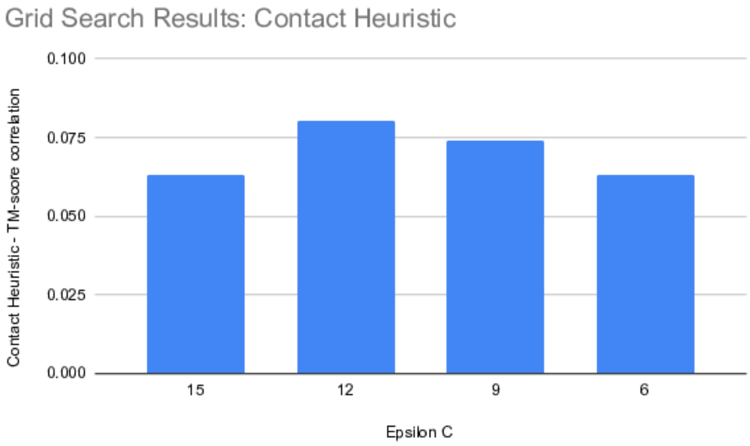

Fig. 2: Grid search over contact score cutoff values shows that $\epsilon_{CS} = 12$ Å provides the greatest correlation between the contact heuristic and TM-score.

2 LoTOP Purification

LoTOP was purified as described in the Materials and Methods. Size exclusion chromatagraphy shows single monomeric species. SDS page gel shows the purity of the NMR samples.

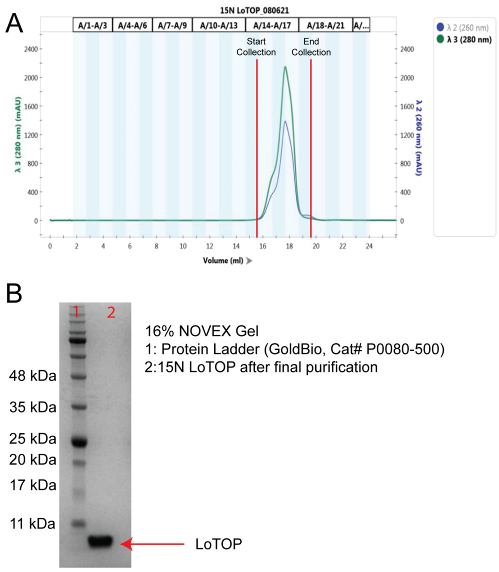

Fig. 3: Purification of LoTOP. A. Size Exclusion Chromatogram of ^{15}N labeled LoTOP showing it to be a single monomeric protein. B. SDS page gel of ^{15}N labeled LoTOP.

3 Design of LoTOP

LoTOP was designed by permuting the secondary structure elements of TOP7 thus simplifying its topology. This is shown in the figure.

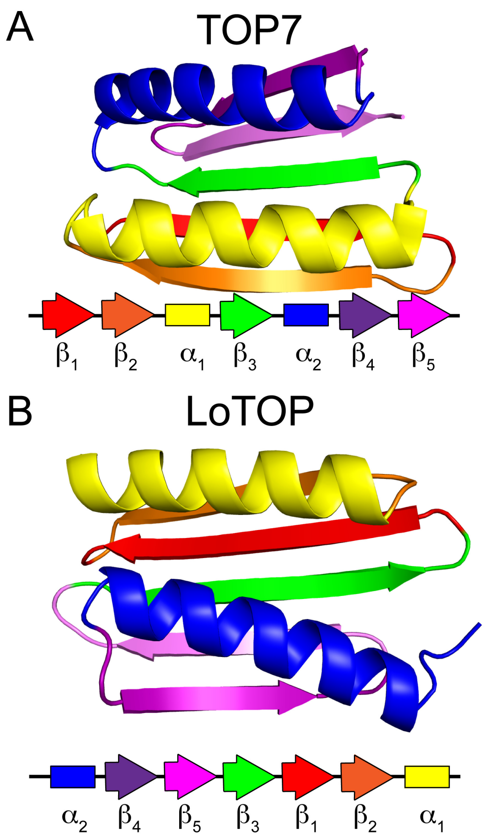

Fig. 4: Top7 and LoTOP structure and topology. A. The structure and topology of TOP7. B. The designed structure and topology of LoTOP.

4 LoTOP Stability

The stabiltiy of LoTOP was determined by measuring the change in the CD spectrum at 227 nm as the denaturant guanidinium hydrochloride was tritrated. The ΔG of unfolding was found to be 12.1±0.4 kcal/mol.

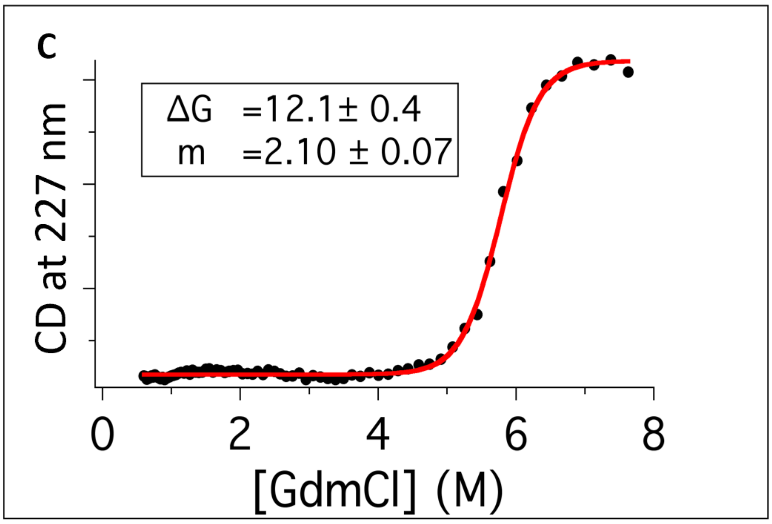

Fig. 5: Guanidinium hydrochloride titration of LoTOP.

5 LoTOP NMR

^{15}N HSQC of LoTOP with HN assignments. Assignments were performed with standard triple resonance techniques.

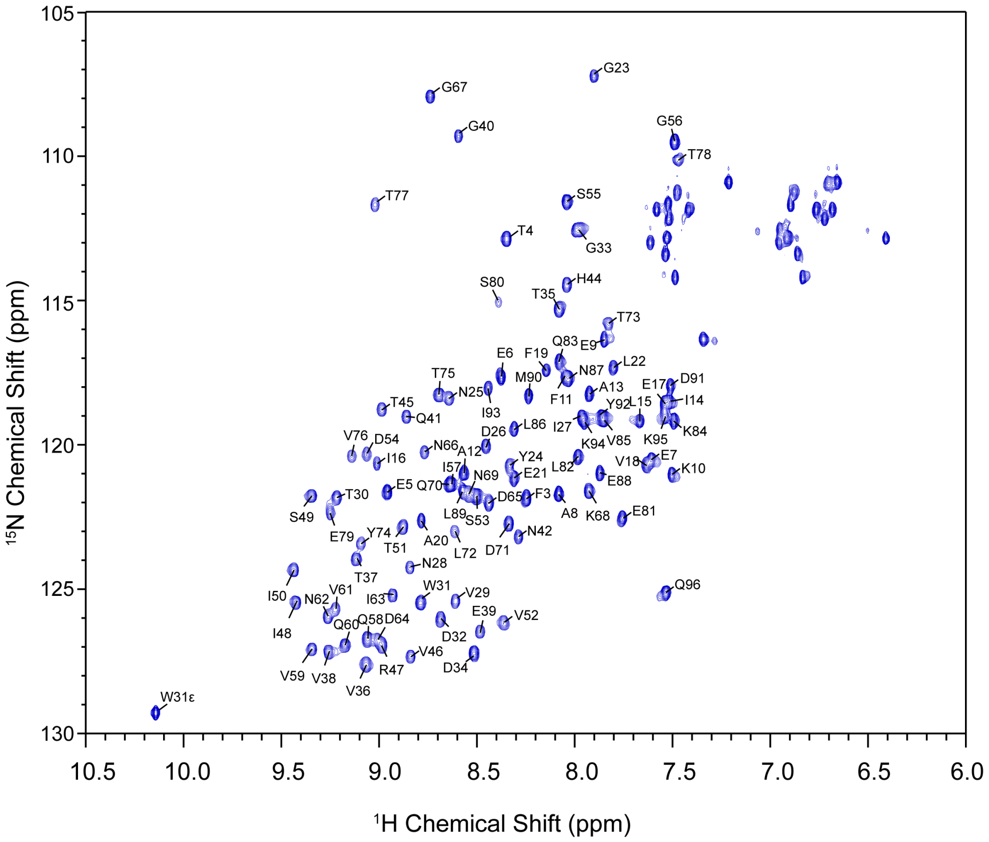

Fig. 6: ^{15}N HSQC of LoTOP with HN assignments
